# Genome-Wide Identification and Functional Validation of the Cold-Tolerance WRKY Transcription Factor in Sweetpotato

**DOI:** 10.3390/plants15193020

**Published:** 2026-10-02

**Authors:** Wenhui Yu, Zhechen Ma, Lukuan Zhao, Yang Liu, Bingqian Gao, Shizhuo Xiao, Rong Jin, Xibin Dai, Yanmei Zhang, Zhilin Zhou, Qinghe Cao

**Affiliations:** 1 Jiangsu Key Laboratory of Phylogenomics and Comparative Genomics, School of Life Sciences and Food Engineering, Jiangsu Normal University, Xuzhou 221131, China; 2 Jiangsu Provincial Key Laboratory for Plant Taxonomy, Resource Conservation and Utilization of Plant Resources, Institute of Botany, Jiangsu Province and Chinese Academy of Sciences, Nanjing 210014, China; 3 Key Laboratory of Biology and Genetic Breeding of Sweetpotato, Ministry of Agriculture and Rural Affairs, Xuzhou Institute of Agricultural Sciences in Jiangsu Xuhuai District, Xuzhou 221131, China

**Keywords:** sweetpotato (*Ipomoea batatas* (L.) Lam.), WRKY transcription factor, cold tolerance

## Abstract

Sweetpotato (*Ipomoea batatas* (L.) Lam.) is a vital global food crop whose production and geographical distribution are severely constrained by low-temperature stress. While WRKY transcription factors are known to regulate plant stress responses, their specific roles in sweetpotato cold tolerance remain largely unexplored. In this study, we conducted a genome-wide identification of the WRKY family in the hexaploid sweetpotato genome, identifying 476 *IbWRKYs* classified into three major groups and seven subgroups. Synteny analyses revealed that whole-genome duplication (WGD)/segmental duplication (SD) was the primary mode of gene duplication for this conserved family. To identify key cold-responsive regulators, we evaluated the cold tolerance phenotypes of 232 diverse accessions and performed comparative transcriptome profiling on representative cold-tolerant and cold sensitive cultivars. Integrated transcriptomic and co-expression network analyses highlighted *IbWRKY460* as a leading cold-responsive candidate. Quantitative RT-PCR confirmed its cold-induced expression, the GFP fusion protein localized to the nucleus, and heterologous expression in yeast provided preliminary evidence of protective activity during low-temperature exposure. Together, these findings prioritize IbWRKY460 for in-planta functional validation and provide a genomic framework for investigating cold-responsive WRKY genes in sweetpotato.

## 1. Introduction

Sweetpotato (*Ipomoea batatas* (L.) Lam.), a member of the Convolvulaceae family, is the seventh most important food crop globally [1]. Native to Central and South America and the Greater and Lesser Antilles, sweetpotato is now extensively cultivated across tropical and subtropical regions worldwide. It is globally recognized as a vital, nutrient-dense crop due to its rich content of dietary fiber, carbohydrates, vitamins, and essential minerals [2]. It is also extensively cultivated across most regions of China, holding great potential for promoting agricultural production and regional economic development. However, sweetpotato is highly sensitive to low temperatures, typically ceasing growth when temperatures drop below 15 °C. Compared to other environmental factors such as drought and high temperatures, cold stress poses a more severe threat, significantly restricting its growth, yield, and geographical distribution. Although successful cultivation in higher latitudes, such as Heilongjiang Province, China, has been reported, its expansion remains constrained by cold stress. The primary symptoms of cold stress in sweetpotato include decreased photosynthetic capacity, phospholipid degradation, altered lipid composition, excessive reactive oxygen species (ROS) accumulation, and compromised cell membrane permeability. These physiological disruptions compromise cellular homeostasis, leading to abnormal growth manifestations such as leaf chlorosis, water loss, and reductions in plant length and weight [3].

In response to biotic and abiotic stresses, plants activate complex signaling networks that ultimately trigger extensive transcriptional reprogramming to execute adaptive responses [4]. Numerous studies have demonstrated that various transcription factor (TF) families, including bHLH (basic helix–loop–helix), bZIP (basic leucine zipper), MYB (myeloblastosis), NAC (NAM, ATAF1/2, and CUC2), and WRKY, synergistically regulate the cold stress signaling network [5,6,7,8,9]. Among these, the WRKY TFs constitute a large family that plays important regulatory roles in biotic and abiotic stress responses, as well as in development, and other physiological processes [10,11,12,13]. The functional core of this protein family is the WRKY domain, which consists of a highly conserved heptapeptide sequence (WRKYGQK) flanked by a C2H2- or C2HC-type zinc finger motif [14]. This structural feature is essential for the recognition and binding to *cis*-acting elements in the promoters of target genes [13]. Based on the number of WRKY domains and the zinc finger motif type, WRKY proteins are classified into three major groups [15]: Group I proteins two WRKY domains with a C2H2 zinc finger; Group II proteins have a single WRKY domain with a C2H2 zinc finger; and Group III proteins possesses a single WRKY domain with a C2HC zinc finger. Furthermore, based on sequence homology, Group II can be further subdivided into five subgroups: IIa–IIe [13,16].

Since the first WRKY gene, SPF1, was cloned from sweetpotato, WRKY transcription factors (TFs) have garnered increasing attention for their diverse functions [17,18]. In the context of cold stress responses, numerous studies have demonstrated the crucial regulatory roles of WRKY proteins across various plant species. For instance, in rice, OsWRKY63 negatively regulates chilling tolerance via the OsWRKY63-OsWRKY76-OsDREB1B transcriptional regulatory cascade [19], whereas OsWRKY71 acts as a transcriptional repressor to enhance it by regulating downstream target genes [20]. Similarly, *CiWRKY31* is induced by cold stress and positively regulates cold tolerance in *Citrus ichangensis* [21]. In *Camellia sinensis*, *CsWRKY2* enhances cold defense by participating in the abscisic acid (ABA) signaling pathway [22]. Furthermore, heterologous overexpression of *TrWRKY79* from white clover and *PmWRKY57* from Prunus mume significantly improves cold tolerance in *Arabidopsis* [9,23]. In tomato, genome-wide identification revealed 81 *WRKY* genes, at least 27 of which are differentially expressed under cold stress [24]. The functions of several of these genes have been experimentally validated. For example, *SlWRKY50* enhances cold tolerance by regulating jasmonic acid (JA) biosynthesis, with the JA signaling pathway in turn mediating *SlWRKY50* expression via the transcriptional activation of *SlMYC2* [25]. SlWRKY33 improves cold tolerance by directly targeting genes encoding kinases, TFs, and molecular chaperones [26], while SlWRKY51 achieves this by promoting proline accumulation [27]. Additionally, SlWRKY54 binds to the *SlDLO2* promoter and interacts with SlZF61 to form a protein complex. This complex synergistically activates *SlDLO2* transcription, thereby improving seedling cold tolerance by promoting chlorophyll synthesis and overall photosynthetic productivity [28]. Several regulators, including IbMPK3 (sweetpotato mitogen-activated protein kinase 3) and IbbHLH116, have been implicated in sweetpotato cold tolerance [29,30]. Nevertheless, the functional characterization of WRKY TFs in this crop remains limited. Although the first plant WRKY gene was originally cloned from sweetpotato [17], subsequent studies have predominantly focused on their roles in anthocyanin accumulation, salt stress, and disease resistance [31,32,33]. To date, IbMPK3/IbMPK6-mediated phosphorylation of the WRKY protein IbSPF1 has been linked to cold tolerance in sweetpotato [34]. However, the genetic function, direct targets, and possible hormone responsiveness of IbWRKY460 remain unknown.

Cold shock domain (CSD) proteins constitute an additional layer of plant cold adaptation. Unlike bacterial cold shock proteins, plant CSD proteins generally contain an N-terminal nucleic-acid-binding CSD together with glycine-rich regions and CCHC zinc fingers, and several members exhibit RNA-chaperone activity [35]. Cold-responsive CSD proteins have been characterized in wheat, rice, and *Arabidopsis*. In *Arabidopsis*, loss of AtCSP3 increases freezing sensitivity, whereas its overexpression enhances freezing tolerance without substantially activating the canonical CBF/DREB1 pathway [36]. These findings support a role for plant CSD proteins in post-transcriptional protection of RNA structure, stability, or translation during cold stress. However, no direct experimental evidence currently links a sweetpotato CSD protein to IbWRKY460.

In this study, we performed a genome-wide identification and phylogenetic analysis to systematically characterize and classify the putative WRKY gene family in sweetpotato. We then evaluated the cold-tolerance phenotypes of different sweetpotato accessions and performed transcriptome sequencing of representative accessions with contrasting cold responses. Integration of differential-expression and co-expression analyses identified cold-responsive WRKY candidates, and IbWRKY460 was further examined by quantitative RT-PCR, subcellular localization, and heterologous expression in yeast. This study provides a genomic framework for investigating WRKY-mediated cold responses in sweetpotato and prioritizes IbWRKY460 for subsequent in-planta functional analysis.

## 2. Results

### 2.1. Genome-Wide Identification and Phylogenetic Classification of the IbWRKY Family

**Figure 1:**
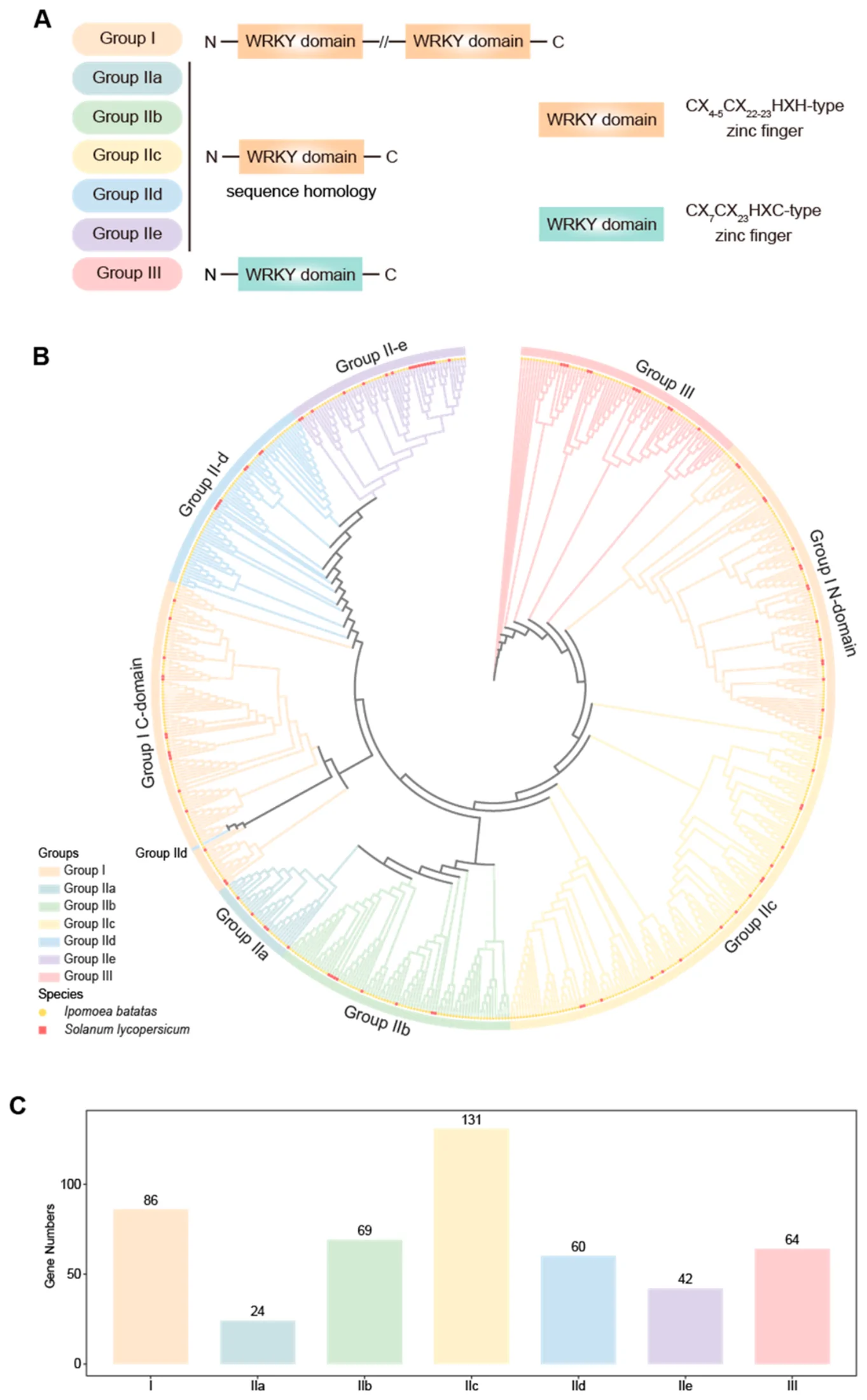
Domain characteristics and phylogenetic analysis of WRKY genes in sweetpotato. (**A**) Schematic representation of domain architectures across different WRKY subfamilies. The diagram illustrates the number of WRKY domains and the types of zinc-finger motifs. (**B**) Phylogenetic tree of sweetpotato (IbWRKY) and tomato (SlWRKY) proteins. The outer colored ring delineates the various WRKY subfamilies (Groups IIII). Yellow circles and red squares indicate WRKY proteins from *Ipomoea batatas* and *Solanum lycopersicum*, respectively. (**C**) Bar chart displaying the number of members in each WRKY subfamily, with bar colors corresponding to their respective subfamilies as shown in (**A**,**B**).

To identify WRKY TFs potentially involved in cold tolerance, we performed a genome-wide screening in sweetpotato, which yielded 476 putative WRKY TFs designated as IbWRKYs (Table S1). Based on the number of WRKY domains and the type of associated zinc finger motifs, these members were initially categorized into three major groups (Figure 1A): Group I (86 members), characterized by two WRKY domains and a C2H2-type zinc finger; Group II (326 members), containing a single WRKY domain with a C2H2-type zinc finger; and Group III (64 members), possessing a single WRKY domain with a C2HC-type zinc finger. Furthermore, based on phylogenetic topology and domain architecture, Group II can be further subdivided into five distinct subgroups (IIa–e).

To elucidate the evolutionary relationships and refine the classification of the IbWRKY family, we constructed a phylogenetic tree using the WRKY domains of all identified IbWRKYs, incorporating representative SlWRKY domains from *Solanum lycopersicum* as evolutionary references (Figure 1B). The phylogenetic topology supported the initial structural grouping and revealed several notable features. Within Group I, the N-terminal and C-terminal WRKY domains formed two distinct lineages, the N-terminal domains clustered with Group III (C2HC-type zinc finger), whereas the C-terminal domains clustered with Group IId (C2H2-type zinc finger). As the predominant subfamily, Group II accounts for approximately 68.3% of all identified IbWRKYs. Among these, Subgroup II-c is the largest, comprising 131 members, followed by II-b (69), II-d (60), and II-e (42), whereas Subgroup II-a is the smallest with only 24 members (Figure 1C). This distribution pattern aligns well with established WRKY categorization models.

### 2.2. Conserved Motif Profiles and Gene Structures Reveal Divergence Among IbWRKY Groups

**Figure 2:**
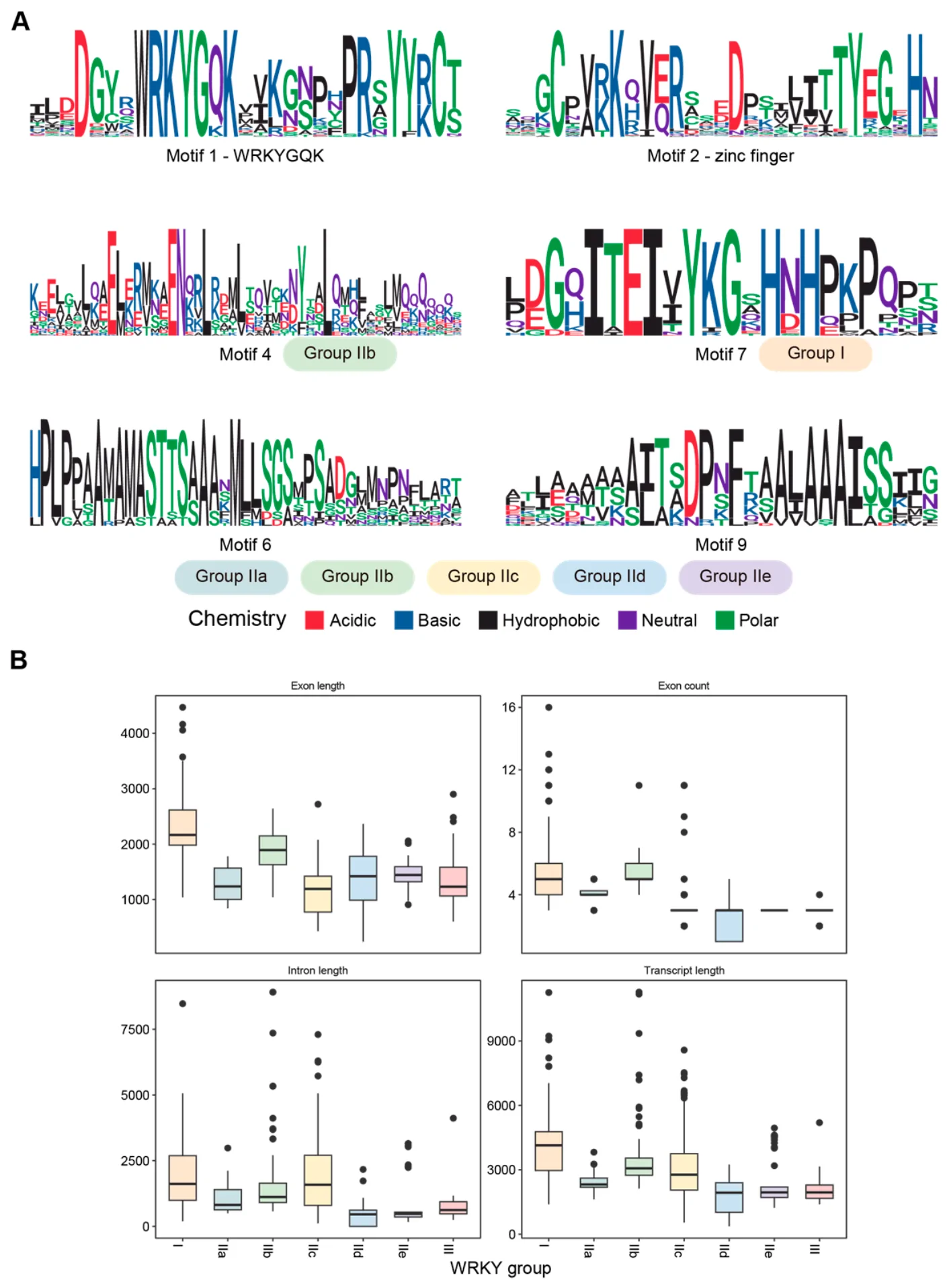
The motif distributions and gene structures of the IbWRKY groups. (**A**) Specific IbWRKY domains were recorded. (**B**) Gene structure attributes of different WRKY groups.

To comprehensively characterize the structural conservation and divergence of the seven IbWRKY subgroups, we analyzed the distribution of conserved motifs across the entire family (Figure 2A). The WRKY domain is canonically defined by a highly conserved heptapeptide sequence, WRKYGQK. Consistent with this, our analysis revealed that Motif 1, which represents the core WRKY domain, predominantly contains the canonical WRKYGQK sequence across the identified IbWRKYs. Notably, we also observed a few natural variants within this motif, specifically WRKYGKK and WRKYGTK. These variants are known to exist in other plant WRKY families and typically retain their DNA-binding capabilities. Furthermore, Motif 2 was identified as the zinc-finger structure (C2H2 or C2HC-type) flanking the WRKY domain, which is indispensable for specific target DNA recognition. The universal presence of these two core functional motifs strongly validates the accuracy and reliability of our genome-wide identification.

Beyond these universally conserved core domains, we identified several group-specific motifs that clearly delineate the structural divergence among the major lineages. Specifically, Motif 7 was found to be exclusively distributed among Group I members. In contrast, Group II proteins were uniquely characterized by the presence of Motif 6 and Motif 9. Furthermore, the motif composition provided additional structural resolution for the sub-classification within Group II (Figure S1). For instance, Motif 4 was identified exclusively in Group IIb, serving as a distinct molecular signature (Figure 2A). Collectively, the distinct distribution patterns of these conserved motifs (Figures S1 and S2) not only corroborate the phylogenetic classification but also highlight the structural conservation and potential functional divergence among different subgroups, providing valuable structural clues for their future functional characterization.

To further elucidate the genomic features of the IbWRKY family, we systematically compared the gene structure attributes, including exon/intron sequence lengths, across seven subgroups (Figure 2B). The results revealed the exon number and length divergence. Among all subgroups, Group I and II-b exhibited higher exon counts, with a median of approximately five exons. Furthermore, Group I contained several genes harboring more than 10 exons (up to 16). In contrast, the exon numbers in Groups IId, IIe, and III were highly conserved, with a median of only 2–3 exons. Regarding the total exon length (coding sequence length), Group I and IIb possessed significantly longer exons (median ~2000 bp) compared to the other five subgroups, which clustered within the 1000–1500 bp. Collectively, these findings suggest that Group I and IIb have tended to maintain more complex gene architectures (characterized by increased exon numbers and extended sequence spans), whereas IId, IIe, and III have evolved towards a simplified and highly conserved structural paradigm.

### 2.3. Chromosomal Distribution and Subgenome Bias of IbWRKY Genes

**Figure 3:**
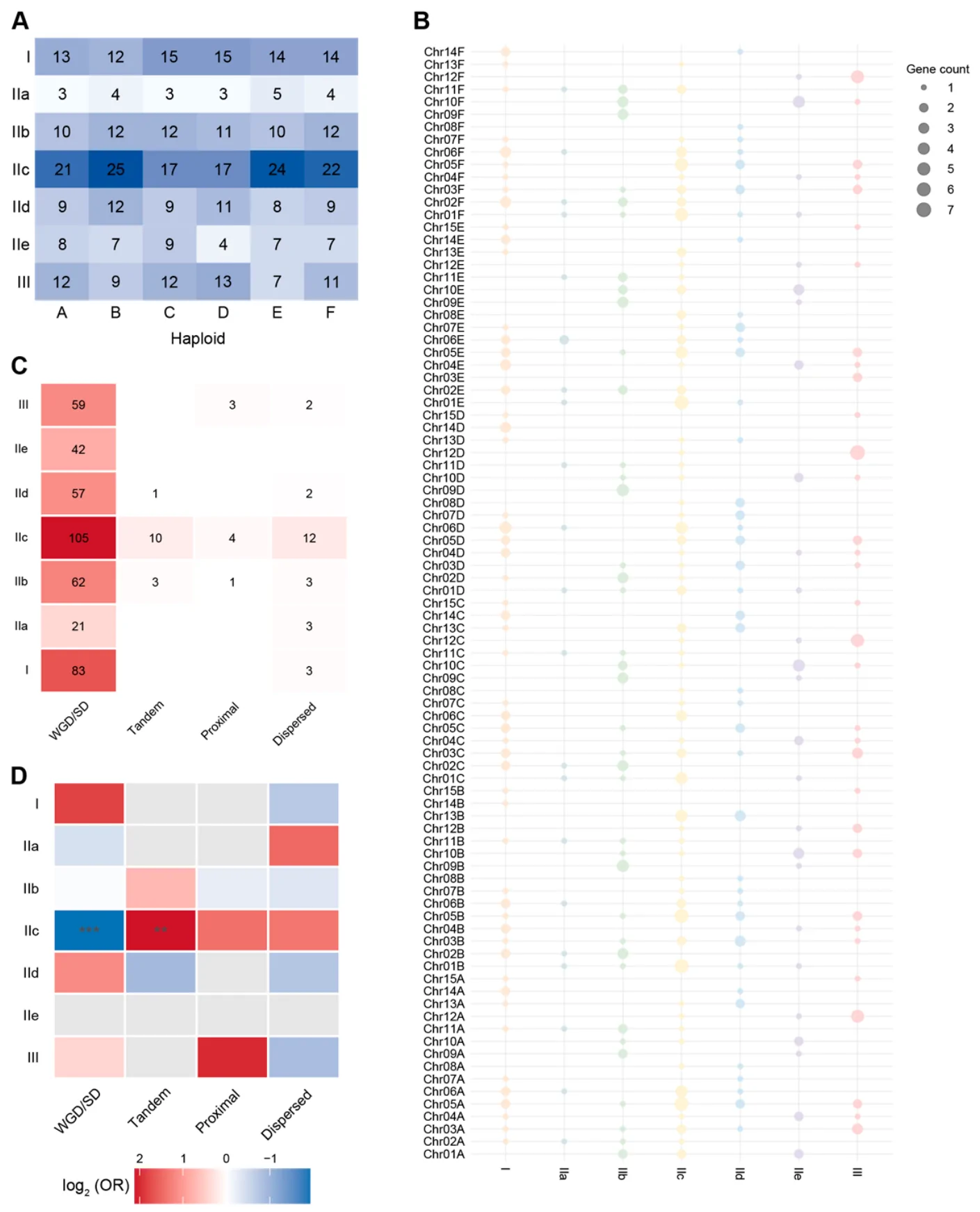
Chromosomal distribution of *IbWRKY* genes. (**A**) The heatmap showed the copy number of different IbWRKY groups across six haploids of the sweetpotato. (**B**) The balloon plot showed the copy number of different IbWRKY groups distributed on each chromosome. (**C**) Heatmap showing the count of *IbWRKY* genes derived from different duplication modes across subfamilies. Numbers and color intensity within cells represent gene counts. Duplication modes include WGD/SD (whole-genome duplication/segmental duplication), Tandem, Proximal, and Dispersed duplication. (**D**) Fisher’s exact test enrichment analysis of duplication modes for each subfamily relative to the whole-family background. The color scale indicates the odds ratio (log_2_(OR)), with red representing enrichment, blue indicating depletion, and gray representing no significant trend or absence of events. Asterisks indicate statistical significance (** *p*-value < 0.01, *** *p*-value < 0.001).

To investigate the chromosomal distribution of IbWRKYs, we mapped all identified *IbWRKY* genes to the sweetpotato reference genome. A total of 462 out of 476 IbWRKYs were successfully anchored onto 89 out of the 90 chromosomes across the six haplotypes (A–F) of the hexaploid sweetpotato (2n = 6x = 90) (Figure 3A). At the haplotype level, the *IbWRKY* genes exhibited a relatively balanced distribution, ranging from 74 members in haplotype D to 81 members in haplotype B, without drastic copy number differences. However, the distribution patterns of specific IbWRKY groups across these haplotypes varied considerably (Figure 3B). For instance, Group IIc, the largest subfamily, showed a pronounced reduction in copy numbers specifically in haplotypes C and D. In contrast, Group I, IIa, and IIb were uniformly distributed across all haplotypes. These results indicate that while the overall copy number of *WRKY* genes remains relatively stable among the six haplotypes of the sweetpotato, the distribution of specific IbWRKY subgroups exhibit heterogeneity.

Furthermore, we analyzed the micro-distribution of IbWRKYs on individual chromosomes (Figure 3B). Notably, no IbWRKY gene was identified on Chr15F, which accounts for the 89 mapped chromosomes. Across the remaining chromosomes, the number of IbWRKYs per chromosome averaged 5.2, with a median of 5. Several chromosomes, including Chr01B, Chr05A, Chr05B, Chr05E, Chr05F, and Chr06D, harbored more than 10 members, approximately double the median value. Conversely, a few chromosomes, such as Chr08F and Chr14B, contained only a single IbWRKY member. These uneven distributions highlight significant copy number variations of *IbWRKYs* across the genome. Regarding group-specific distribution, while certain groups were entirely absent on specific chromosomes, most chromosomes harbored 1 to 6 members of a given group. Interestingly, Group IIc exhibited a distinct clustering pattern, with six copies present on each of Chr01B, Chr01E, Chr05A, and Chr05B, suggesting a preferential enrichment of this group on Chr01 and Chr05. These observations suggest that during the formation of the sweetpotato allohexaploid, structural divergence may have led to subtle copy number variations among different haplotypes. Nevertheless, for the vast majority of the gene family, such bias is not overtly prominent, indicating that the IbWRKY family has maintained a relatively balanced and stable retention pattern across the subgenomes.

To elucidate the mechanisms driving the copy number variation of the *IbWRKY* gene distributed on chromosomes, we classified the identified genes based on their duplication modes (Table S2). Our analysis revealed that whole-genome duplication (WGD) or segmental duplication (SD) served as the primary driving force for the expansion of the IbWRKY family, dominating across all major groups (Figure 3C). Specifically, within Group IIc, the largest subfamily, up to 105 genes were retained via WGD/SD. This was followed by Group I (82 genes) and IIb (62 genes). Even in the smallest Group IIa, 21 genes originated from WGD/SD events. This overwhelming prevalence of WGD/SD-derived genes reflects the extensive synteny retained among the subgenomes of the allohexaploid sweetpotato. In contrast, tandem, proximal, and dispersed duplications were relatively rare across the family. For instance, Group I and IIa each contained only a single dispersed gene, with the remainder being WGD-derived; Group IIe consisted entirely of WGD/SD-derived genes. Furthermore, tandem duplications were exclusively restricted to Group IIb, IIc, and IId. The dominance of WGD/SD, driven by the massive retention of collinear signals post-polyploidization, coupled with the scarcity of other duplication types, suggests that the IbWRKY family is conserved and has not undergone massive non-WGD/SD-mediated expansion or extensive gene loss.

To determine whether specific subgroups exhibited distinct preferences for certain duplication modes, we performed an enrichment analysis using Fisher’s exact test, comparing the duplication profile of each group against the background of the entire IbWRKY family (Figure 3D, Table S3). Notably, Group IIc exhibited a highly significant depletion in the WGD mode, while demonstrating a highly significant enrichment in tandem duplications. Although Group IIc possessed the highest absolute number of WGD genes (105) and only 10 tandem-duplicated genes, its relative proportion of tandem duplications was significantly higher than the family-wide background. This paradox indicates that, during post-polyploidization evolution, Group IIc preferentially utilized tandem duplication for localized expansion, exhibiting a significantly lower relative dependency on WGD/SD compared to the family average. Additionally, other subgroups displayed subtle, albeit non-significant, enrichment trends. For instance, Group I and IId showed a tendency towards WGD enrichment, while Group III exhibited a slight preference for proximal duplication. Conversely, Groups I and IIb showed a tendency towards depletion in dispersed duplications.

### 2.4. Phenotypic Evaluation of Cold Tolerance and Transcriptome Sequencing of Representative Accessions

To further screen for sweetpotato cultivars with strong cold tolerance, we evaluated a diverse panel of 232 accessions collected from various geographical origins (Table S4). Based on their comprehensive cold-tolerance phenotype scores (CTPS), which ranged from −1 to 8, the accessions were categorized into three distinct phenotypic groups: cold-tolerant (R), moderately cold-tolerant (M), and cold-sensitive (N). Phenotypic evaluation revealed that 182 and 20 accessions were classified into the N and M groups, respectively. Notably, accessions in both of these groups completely failed to initiate new root growth under cold stress, accounting for 87.1% of the total evaluated germplasm. This high proportion indicates that the rooting capacity of the vast majority of sweetpotato accessions is severely inhibited by low-temperature stress. In contrast, we successfully identified 30 cold-tolerant accessions (group R) that maintained normal rooting capacity under the same cold stress conditions.

To investigate the transcriptional reprogramming mechanisms underlying these phenotypic divergences, one representative accession from each group, CX55 (‘Ji10216’, R), CX36 (‘Xu17-10’, M), and CX65 (‘LongShu601’, N), was selected for transcriptome profiling. The transcriptional responses of their leaves (L) and tuberous roots (T) were examined under cold stress. Clean RNA-seq reads from these samples were mapped to the hexaploid sweetpotato reference genome (cv. Beauregard, see Section 4). The resulting gene expression matrix was subsequently subjected to principal component analysis (PCA) and Pearson correlation analysis (Figure S3). The results demonstrated clear transcriptomic separation among the 12 distinct experimental groups (comprising three accessions, two tissue types, and two time points). This distinct clustering confirms the occurrence of tissue-specific transcriptional reprogramming in response to cold stress. Furthermore, the high reproducibility and robustness of the transcriptomic data ensure its reliability for downstream differential expression and functional analyses.

### 2.5. Transcriptomic Reprogramming and Functional Enrichment Analysis Under Cold Stress

**Figure 4:**
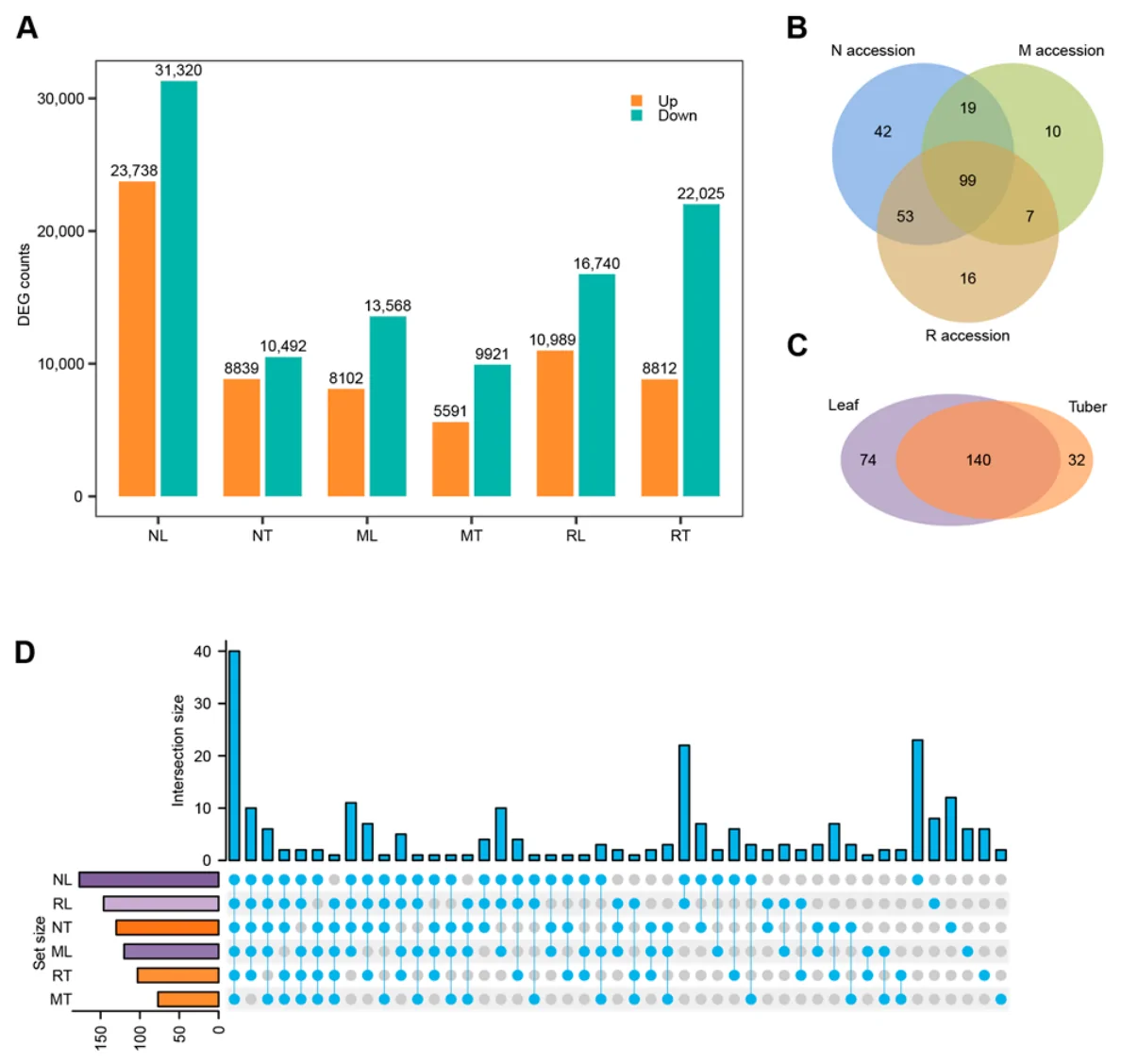
Transcriptomic landscape of cold stress response and intersection analysis of differentially expressed IbWRKY genes. (**A**) Numbers of up- and downregulated DEGs across six comparison groups in sweetpotato under cold stress. (**B**–**D**) Overlap analyses of cold-responsive IbWRKY genes across different accessions ((**B**), Venn diagram), tissues ((**C**), Venn diagram), and all six treatment combinations ((**D**), UpSet plot).

To systematically evaluate the transcriptomic differences among different sweetpotato accessions and tissues under cold stress, we first identified and analyzed differentially expressed genes (DEGs) across the six experimental groups (Figure 4A). A total of 170,137 DEGs were identified across all comparisons. Notably, the number of downregulated genes consistently outnumbered that of upregulated genes among all samples (Figure 4A). The highest number of DEGs was observed in the leaves of the cold-sensitive N accession, whereas the lowest was found in the tuberous roots of the moderately tolerant M accession, potentially suggesting that the leaves of the N accession underwent the most extensive transcriptional reprogramming. By extracting the IbWRKY family members from the complete DEG datasets, we identified 246 IbWRKY TFs that were differentially expressed across various samples (Table S5). Among these, 21 IbWRKYs exhibited divergent expression trends depending on the sample and treatment. Of the remaining IbWRKYs, 177 were consistently upregulated across all treatments where they were expressed, while 48 were universally downregulated.

To further systematically evaluate the potential response of the IbWRKY family to cold stress, we analyzed the intersections of DEGs among different samples (Figure 4B–D). We found that 99 IbWRKYs exhibited transcriptional changes in response to cold stress across all three accessions (Figure 4B), indicating that these genes likely serve as key regulatory components in the cold tolerance processes of different sweetpotato genotypes. Meanwhile, 16 IbWRKYs were identified as specifically responsive in the cold-tolerant R accession, potentially participating in the signaling pathways unique to the cold-tolerant phenotype. Furthermore, we examined the tissue-specific expression patterns of IbWRKYs (Figure 4C). The results showed that 140 IbWRKYs (56.9%) shared common transcriptional response features in both leaves and tuberous roots. Specifically, 74 IbWRKYs were uniquely responsive in leaves, while only 32 were specific to tuberous roots. A joint analysis of the six distinct phenotypic combinations revealed that 40 IbWRKY genes exhibited significant transcriptional changes across all six treatment groups (Figure 4D), suggesting that these 40 IbWRKYs may act as conserved transcriptional regulators during the transcriptional reprogramming of the cold stress response.

### 2.6. Identification of Core Co-Expression Modules and Candidate Cold-Responsive IbWRKYs

**Figure 5:**
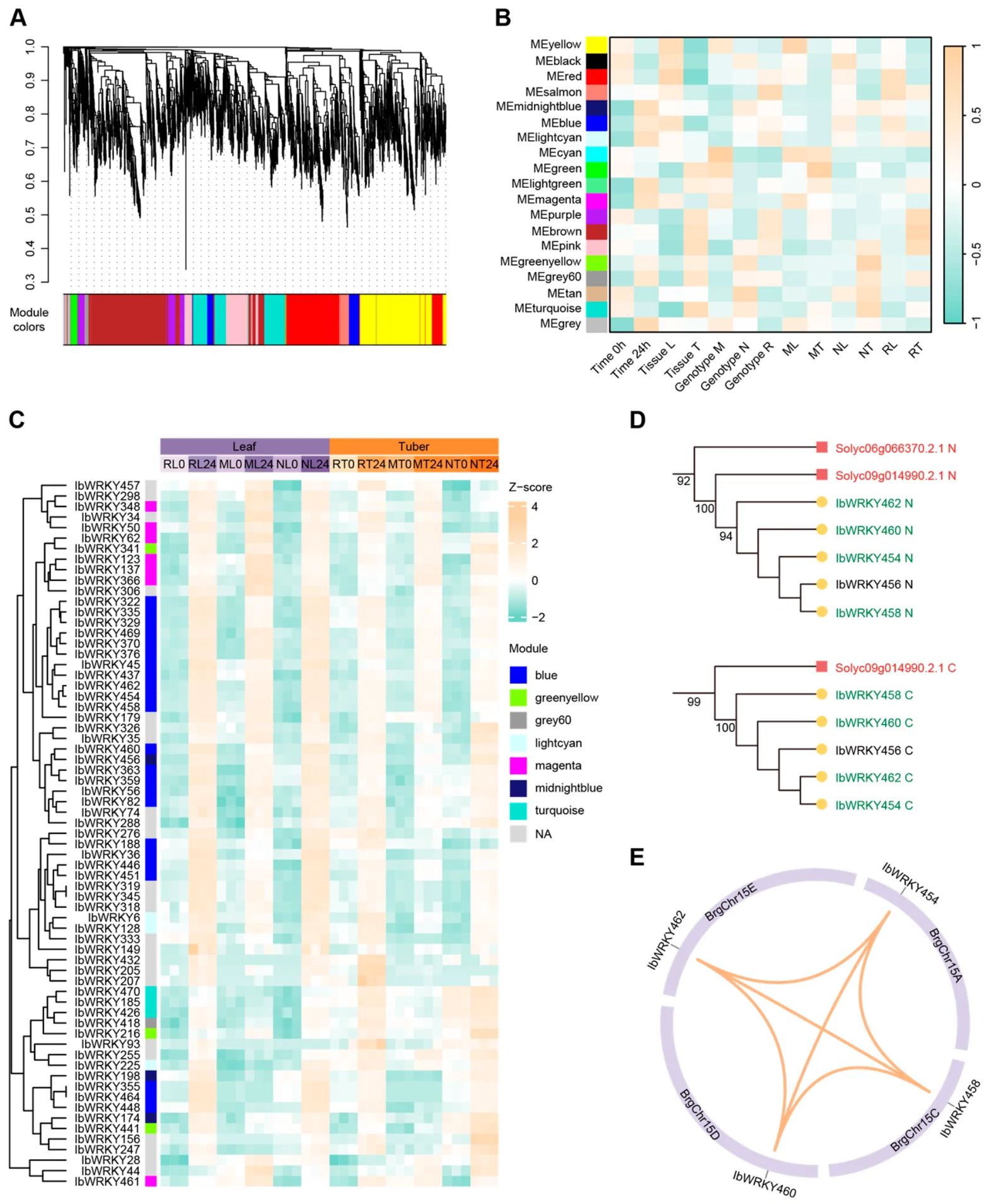
Weighted gene co-expression network analysis (WGCNA) and identification of the core cold-tolerance candidate gene *IbWRKY460* in sweetpotato. (**A**) Gene clustering dendrogram and module colors. The hierarchical clustering dendrogram shows the co-expression relationships among 10,000 genes based on their expression patterns across 36 samples. The color bands beneath the dendrogram represent the module assignment of each gene, where genes with similar expression patterns are clustered into the same module. Modules are identified by different colors using the dynamic tree cut algorithm with a minimum module size of 30 genes. The grey module typically contains genes not assigned to any specific module. The scale-free topology parameter (power) used for network construction was 24, with a merge cut height of 0.25 for module merging. (**B**) Module-trait correlation heatmap. The heatmap displays Pearson correlation coefficients between module eigengenes (rows) and experimental traits (columns). Each cell shows the correlation coefficient (top number) and corresponding *p*-value in parentheses (bottom). (**C**) Expression heatmap of 67 cold-induced upregulated IbWRKY transcription factors across samples. (**D**) Phylogenetic analysis of N-terminal (top) and C-terminal (bottom) WRKY domains from four candidate IbWRKY proteins alongside cold-responsive tomato (Solyc) homologs. (**E**) Synteny circle plot showing the collinearity of key IbWRKY candidate genes across subgenomes of chromosome 15 (15A, 15B, 15C, 15D, and 15E).

To systematically investigate the association between gene co-expression networks and cold tolerance phenotypes in sweetpotato, we performed weighted gene co-expression network analysis (WGCNA) using the reference-based transcriptomic data. A total of 19 distinct co-expression modules were identified (Figure 5A). Subsequently, we correlated the module eigengenes with cold treatment durations (0 h and 24 h), tissue type (leaf [L] and tuberous root [T]), genotype (N/M/R), and their combinations (ML, MT, NL, NT, RL, and RT) (Figure 5B, Table S6). The results showed that several modules, including midnightblue, blue, lightcyan, lightgreen, magenta, grey60, and grey, were significantly positively correlated with the 24-h cold stress treatment. Notably, the blue and lightcyan modules also exhibited significant positive correlations with the leaves and tuberous roots of the cold-tolerant accession (RL and RT), respectively (Figure 5B, Table S7). This suggests that genes within these modules may play crucial roles in protecting sweetpotato against cold damage.

To further pinpoint the core module based on known molecular markers, we aligned the amino acid sequences of previously reported core cold-responsive proteins, IbMPK3 [29,34] and IbSPF1 [17,34], against the sweetpotato reference genome. The five homologs of IbMPK3 were distributed in the blue (2), green-yellow (2), and magenta (1) modules. Meanwhile, the five homologs of IbSPF1 were distributed in the blue (4) and green-yellow (1) modules. Based on the strong phenotype-module correlations and the high enrichment of known stress marker genes, we deduced that the MEblue module is highly involved in the signaling pathways under cold stress and likely contains key upstream and downstream regulators. Therefore, we propose that the MEblue module is the core co-expression module driving the cold stress response and determining the divergence in cold tolerance in sweetpotato.

Based on the transcriptional upregulation in both leaves and tuberous roots after cold treatment, we screened 67 IbWRKY transcription factors that potentially participate in cold stress in the cold-tolerant (R) accession (Figure 5C). By analyzing the intersection of these 67 IbWRKYs across different samples, we found that 22 of them belonged to the previously identified MEblue module (Figure 5C). Combining the expression profiles with the core WGCNA module, these 22 MEblue-associated IbWRKYs are likely involved in the cold tolerance signaling pathway. Previous studies have demonstrated that three SlWRKY genes [27,37,38] are involved in the cold tolerance signaling pathway in tomato (Figure 5D). Integrating phylogenetic analysis, we identified the IbWRKYs most closely related to these three SlWRKYs: IbWRKY462, IbWRKY460, IbWRKY454, and IbWRKY458 (Figure 5D). Furthermore, these four IbWRKY genes are located in syntenic regions on chromosome 15 across different haplotypes (Figure 5E). This indicates that they are likely homeologs retained after potential ancestral whole-genome duplication (WGD) [39,40] and may share similar functions. Consequently, these four IbWRKYs are strong candidates participating in the cold tolerance signaling pathway, acting as a potential regulatory module to confer the cold-tolerant phenotype in the sweetpotato.

### 2.7. Functional Characterization of IbWRKY460 in Cold Tolerance

**Figure 6:**
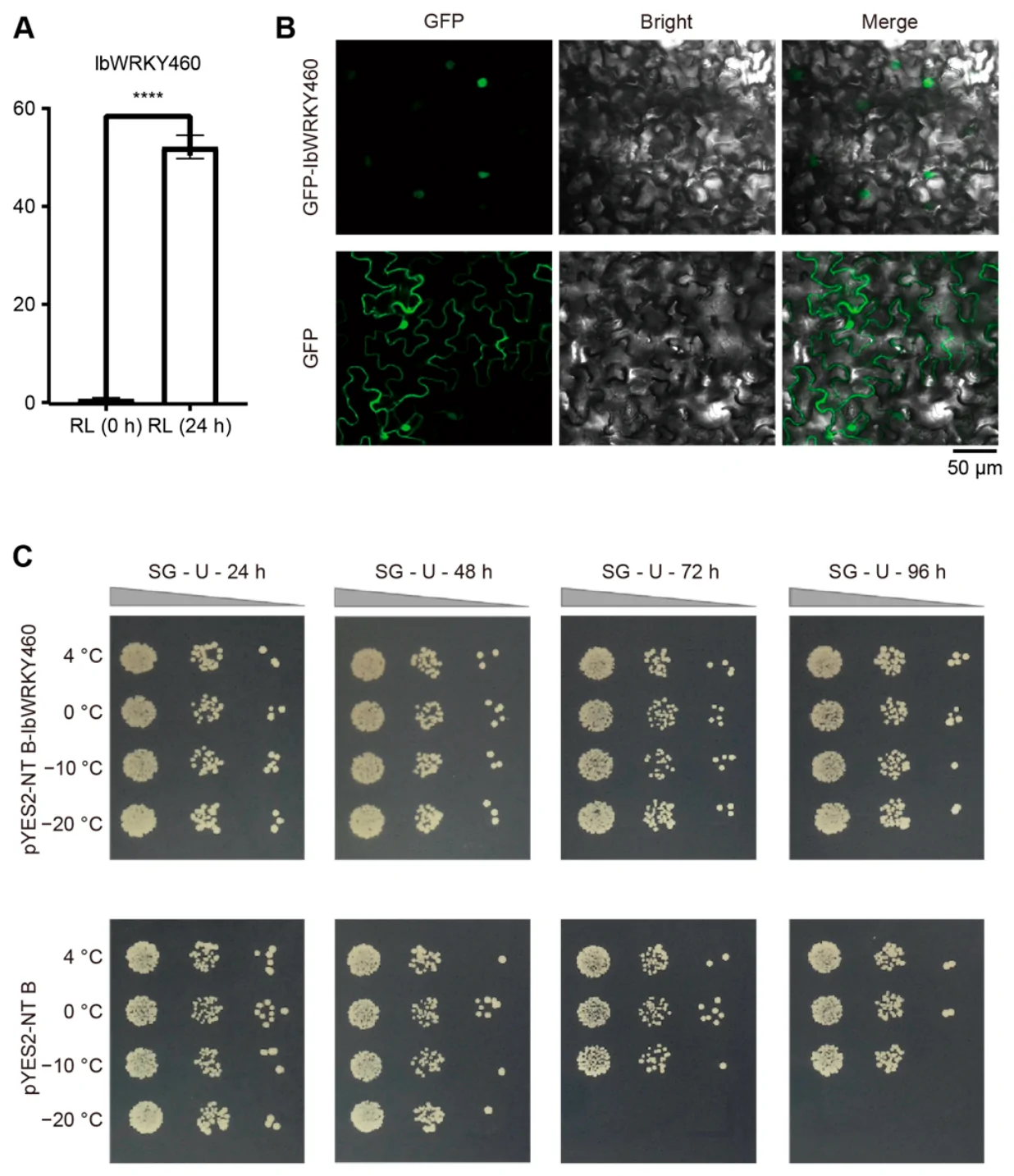
Subcellular localization of IbWRKY460 and its heterologous expression conferring cold tolerance in yeast. (**A**) qRT-PCR of the *IbWRKY460* genes. Data are presented as the mean ± SD (n = 3 biological replicates). **** *p*-value < 0.0001 (two-tailed Student’s *t*-test). (**B**) Subcellular localization of IbWRKY460 in *Nicotiana benthamiana* leaf epidermal cells. GFP-WRKY460 fusion protein and empty vector GFP control (pBIN-GFP2) were transiently expressed in N. benthamiana leaves via Agrobacterium-mediated transformation. Columns from left to right represent green fluorescence signal (GFP), bright field (Bright), and merged channels (Merge). Scale bar = 50 μm. (**C**) Growth and survival assay of recombinant yeast expressing IbWRKY460 under various low-temperature conditions. Yeast strains transformed with pYES2-NT B-WRKY460_CDS or the empty vector control (pYES2-NT B) were exposed to 4 °C, 0 °C, −10 °C, and −20 °C for 24 h, 48 h, 72 h, and 96 h, respectively. Serial 10-fold dilutions 10^0^, 10^−1^, and 10^−2^, indicated by gray triangles) were spotted onto SG-Ura (SG-U) plates and incubated at 30 °C for 3–4 days to document colony growth.

Through reference-based transcriptomic profiling, we chose one of the four candidate WRKY TFs IbWRKY460 as a key candidate gene exhibiting pronounced cold-induced upregulation in the CX55 accession. To validate the expression of this orthologous WRKY locus in the cold-tolerant background, qRT-PCR was performed in RL group using gene-specific primers (Table S8). The results confirmed that the transcript levels of this gene were significantly elevated following cold treatment (Figure 6A), which is highly consistent with the transcriptomic data and suggests its active participation in the cold stress response.

To determine the subcellular localization of the IbWRKY460 protein, the pBIN-GFP2-IbWRKY460 fusion construct and the empty vector control (pBIN-GFP2) were transiently expressed in *Nicotiana benthamiana* leaves via Agrobacterium-mediated infiltration (Figure 6B). Confocal microscopy revealed that the GFP2 control protein emitted green fluorescence in both the nucleus and the cytoplasm, reflecting its free diffusion. In contrast, the GFP2-IbWRKY460 fusion protein was exclusively localized to the nucleus. This nuclear localization aligns with the canonical role of WRKY TFs as nuclear-localized DNA-binding proteins [16].

To further investigate whether the overexpression of this WRKY factor can confer cold tolerance at the cellular level, a heterologous functional assay was conducted in the yeast system. The pYES2-NT B-IbWRKY460 construct and the empty vector control (pYES2-NT B-EV) were transformed into the yeast strain BY4741, and the transformants were subjected to cold treatments at 4 °C, 0 °C, −10 °C, and −20 °C for 24, 48, 72, and 96 h, followed by a serial dilution spotting assay (Figure 6C). Under mild cold stress (4 °C and 0 °C), both the control and IbWRKY460-expressing yeast cells grew normally within 48 h. However, when the cold treatment was extended to 72–96 h, the control yeast cells failed to survive at all tested temperatures. Notably, the yeast cells overexpressing IbWRKY460 maintained robust growth and viability even after prolonged cold exposure, including the most severe treatment at −20 °C. These results provide preliminary functional evidence that the overexpression of this WRKY transcription factor can enhance cold tolerance in a eukaryotic system, potentially explaining how the CX55 accession maintains rooting capacity under low-temperature stress.

## 3. Discussion

Sweetpotato production is increasingly challenged by low-temperature stress, particularly as cultivation expands toward higher latitudes. Here, genome-wide analysis of the hexaploid sweetpotato genome identified an expanded WRKY family, and integration of cold-response transcriptomes with co-expression analysis prioritized IbWRKY460 as a leading cold-responsive candidate. The expression, localization, and heterologous functional data support further investigation of this gene for further study, but they do not yet establish IbWRKY460 as a key positive regulator in sweetpotato plants or define its downstream mechanism.

The 476 IbWRKY loci identified here substantially exceed the WRKY counts reported for diploid tomato and *Arabidopsis* [16,38]. This expansion is consistent with the hexaploid structure and haplotype-resolved organization of the cultivated sweetpotato genome [39,40]. Our synteny analysis further indicated that whole-genome duplication (WGD)/segmental duplication (SD) was the predominant duplication mode within the IbWRKY family. The uneven distribution of IbWRKY genes across homoeologous chromosomes and subgroup-specific motifs may reflect subsequent retention and functional divergence, although these possibilities require direct functional testing. An earlier survey based on the Taizhong6 genome reported 84 IbWRKY genes [41]. The difference from the 476 loci identified here likely reflects differences in cultivar, and the explicit representation of six haplotypes.

Phenotypic evaluation of 232 accessions revealed substantial variation in cold tolerance within sweetpotato. The cold-sensitive accession showed a broader transcriptional response in leaves than the cold-tolerant accession. This pattern may partly reflect stronger cellular perturbation or injury rather than a more effective adaptive response. Conversely, the comparatively restricted response of the cold-tolerant accession, including extensive transcriptional repression in tuberous roots, may be consistent with resource conservation and selective activation of protective pathways. These interpretations remain hypotheses because transcript abundance alone does not distinguish adaptive regulation from secondary consequences of stress injury.

WGCNA identified the blue module as strongly associated with the cold-tolerance traits. The presence of homologs of previously characterized cold-responsive genes, including IbSPF1 and IbMPK3, supports the biological relevance of this module [29,34]. However, co-expression indicates association, not direct regulation; accordingly, IbWRKY460 should be described as a hub-associated candidate until its direct targets and genetic effects are demonstrated in sweetpotato.

*IbWRKY460* (Ibat.Brg.15D_G008070.1) emerged as a leading candidate because of its preferential cold induction in a tolerant genotype, membership in the cold-correlated blue module, and phylogenetic proximity to cold-responsive tomato WRKYs. Its nuclear localization is consistent with, but does not by itself demonstrate, transcription-factor activity. Heterologous expression of *IbWRKY460* improved yeast growth after prolonged low-temperature exposure, providing preliminary evidence of protective activity in a eukaryotic cellular system. Nevertheless, yeast lacks the plant-specific promoters, hormone signaling, tissue context, and developmental regulation required to infer the genetic function of *IbWRKY460* in sweetpotato. The present results therefore do not support describing *IbWRKY460* as versatile, as a proven positive regulator, or as ready for direct use in breeding. A precise conclusion is that *IbWRKY460* is a prioritized cold-responsive candidate whose in-planta function remains to be established.

Plant CSD proteins and WRKY TFs may contribute to cold adaptation at different regulatory levels. CSD proteins bind nucleic acids and can act as RNA chaperones, whereas WRKY proteins are sequence-specific transcriptional regulators [35]. AtCSP3 enhances freezing tolerance without requiring activation of the canonical CBF/DREB1 pathway, illustrating that CSD-mediated protection need not be downstream of a known transcriptional cascade [36]. No direct evidence currently links IbWRKY460 to a sweetpotato CSD protein. Consequently, any CSD-IbWRKY460 relationship should be presented as a testable hypothesis rather than an established regulatory connection.

The downstream mechanism of IbWRKY460 remains unresolved. In tomato, SlWRKY50 promotes chilling tolerance through jasmonic acid (JA) biosynthesis and participates in feedback with JA signaling, whereas SlWRKY54 functions with SlZF61 to activate SlDLO2 and support chlorophyll accumulation and photosynthetic performance during cold stress [25,28]. The phylogenetic proximity of IbWRKY460 to cold-responsive tomato WRKYs supports prioritizing these pathways for comparison, but sequence relatedness alone does not demonstrate conserved targets or mechanisms. The four collinear IbWRKY460-related loci on chromosome 15 homoeologs also raise the possibility of dosage effects or functional redundancy, which should be resolved before assigning a specific mechanism to IbWRKY460. The scarcity of functional studies on IbWRKY genes in cold tolerance, despite the historical discovery of SPF1 in sweetpotato, highlights a critical gap in our understanding of this crop’s stress biology [17]. This study helps narrow that gap by prioritizing IbWRKY460 as a candidate for further functional investigation. However, its value for molecular breeding cannot be assessed until stable gain—and loss-of-function studies in sweetpotato establish its effects on cold tolerance and agronomic performance. The comprehensive classification and chromosomal mapping of the 476 IbWRKY members also provide a foundation for future studies of WRKY-mediated responses to abiotic and biotic stresses.

Phytohormone regulation provides a plausible direction for future investigation. Selected sweetpotato WRKY genes respond transcriptionally to abscisic acid (ABA), salicylic acid (SA), jasmonic acid (JA), and 1-aminocyclopropane-1-carboxylic acid (ACC), although the genes examined in that study are not equivalent to IbWRKY460 [41]. In tomato, SlWRKY50 and JA signaling form a regulatory relationship that contributes to chilling tolerance [25]. In sweetpotato, IbbHLH129 is induced by GA3, methyl jasmonate, and indole-3-acetic acid, repressed by ABA, and enhances short-term cold tolerance by activating the auxin- and gibberellin-related genes IbYUCCA2 and IbGID1 [42]. Together, these findings support examining whether selected growth regulators alter IbWRKY460 expression or its associated cold-response network. They do not, however, demonstrate that exogenous phytohormones activate IbWRKY460 or improve sweetpotato cold tolerance through this factor. Accordingly, phytohormone regulation might be a future research direction.

In conclusion, polyploidization has contributed to extensive expansion of the sweetpotato WRKY family. Multi-layer transcriptomic analysis, nuclear localization, and heterologous yeast assays identify IbWRKY460 as a strong cold-responsive candidate with preliminary protective activity. Its function in sweetpotato, direct downstream targets, possible relationship with CSD proteins, and responsiveness to phytohormones remain open, experimentally testable questions. Resolving these questions will determine whether IbWRKY460 can ultimately be used to improve sweetpotato cold tolerance.

## 4. Materials and Methods

### 4.1. Plant Materials and Genome Resources

For genome-wide identification and bioinformatic analyses, the reference genome assembly and corresponding gene annotation of sweetpotato (*Ipomoea batatas* (L.) Lam. cv. Beauregard, Version 1) were retrieved from the Sweetpotato Genomics Resource (https://sweetpotato.uga.edu/).

To evaluate cold tolerance, a panel of 232 sweetpotato accessions obtained from the National Sweetpotato Genebank in Vitro (Xuzhou, China), including improved cultivars and traditional landraces, was used for phenotypic evaluation (Table S4). Plants were propagated by stem cuttings and planted in plastic pots containing coconut coir, vermiculite, and perlite (5:4:1, *v*/*v*/*v*). Plants were maintained in a controlled-environment growth room at 25 ± 2 °C and 60–70% relative humidity under a 16-h-light/8-h-dark photoperiod with a light intensity of approximately 1000 lx. Plants were watered every 2–3 d to maintain adequate substrate moisture and supplied with half-strength Hoagland nutrient solution once per week. Cold treatment was initiated one week after planting.

### 4.2. Identification of the IbWRKY Gene Family

To identify potential WRKY proteins in sweetpotato, an HMMsearch was performed against the sweetpotato proteome using HMMER (v3.4, http://hmmer.org/) with the WRKY domain HMM (Accession: PF03106) from the Pfam database (Pfam release 38.1) [43,44]. The candidate sequences were further verified using the Batch CD-Search service (https://www.ncbi.nlm.nih.gov/Structure/cdd/wrpsb.cgi, accessed on 24 November 2025) against the NCBI Conserved Domain Database (CDD v3.21). Only sequences with a confirmed WRKY domain in the CDD were retained. All searches were conducted using default parameters. The WRKY domains were then extracted based on the HMMscan results. For members containing two WRKY domains (Group I), the domains were defined as N-terminal and C-terminal WRKY domains according to their positions in the protein sequences.

### 4.3. Phylogenetic Analysis

The WRKY domain sequences of all identified IbWRKYs were combined with those of the SlWRKY family from tomato [38] for phylogenetic analysis. Multiple sequence alignment (MSA) of the WRKY domains was generated using MUSCLE (v5.2) [45] with default parameters. A phylogenetic tree was constructed using the maximum likelihood (ML) method implemented in IQ-TREE (v3.0.1) [46], with nodal support assessed by 1000 bootstrap replicates. The tree was visualized and annotated using the online platform iTOL (v7). The identical pipeline was applied to construct a phylogenetic tree based on the full-length IbWRKY protein sequences.

### 4.4. Conserved Motif Prediction

To predict the conserved motifs of all identified IbWRKY transcription factors, the online tool MEME (v5.5.9, Multiple Expectation Maximization for Motif Elicitation; https://meme-suite.org/meme/tools/meme, accessed on 24 November 2025) was utilized. The maximum number of motifs was set to 10, and all other parameters were kept at their default values. Subsequently, the distribution of CDD domains and predicted motifs, combined with the phylogenetic tree of the full-length IbWRKY sequences, were visualized using the Gene Structure View module in TBtools-II (v2.390) [47] (Table S1).

### 4.5. Gene Structure Characterization and Chromosomal Distribution of IbWRKY Genes

To characterize the gene structures of the IbWRKY family, the GFF3 annotation file of the sweetpotato genome was processed using gffread (v0.12.7) [48]. This allowed for the extraction of the exon number, exon length, intron length, and total transcript length for each of the 476 IbWRKY genes. Furthermore, chromosomal location information was extracted from the GFF3 file to determine the physical distribution of these genes. Of the 476 genes, 462 were successfully mapped to the chromosomes across the different haplotypes (A–F), while the remaining 14 genes were located on unassembled contigs or scaffolds.

### 4.6. Gene Duplication Analysis of IbWRKYs

To identify homologous gene pairs within the sweetpotato genome, an all-to-all protein sequence comparison was performed using BLASTP (v2.12.0+, https://blast.ncbi.nlm.nih.gov/Blast.cgi, accessed on 24 November 2025) with an E-value threshold of 1 × 10^−5^. Subsequently, collinear blocks were inferred using MCScanX (v1.0.0) [49] based on the BLAST alignment results and the genomic positional information. The built-in scripts of MCScanX were then utilized to classify the gene duplication modes.

To determine whether specific IbWRKY groups exhibited a preference for enrichment or depletion via particular duplication mechanisms, the odds ratio (OR) was calculated by comparing the duplication profile of each group against that of the entire IbWRKY family. Fisher’s exact test was employed to evaluate the statistical significance, and the raw *p*-values were corrected for multiple testing using the Benjamini–Hochberg (BH) method. The significance of the enrichment or depletion for specific duplication modes within each group was determined based on these adjusted *p*-values.

### 4.7. Phenotypic Evaluation and Classification of Cold Tolerance in 232 Sweetpotato Accessions

To quantitatively evaluate cold tolerance, the 232 sweetpotato accessions listed in Table S4 were propagated and cultivated under the conditions described in Section 4.1. Cold treatment at 4 °C was initiated one week after planting and continued for 20 d. For each accession, nine plants were arranged in three pots, with three plants per pot; each pot was considered an independent biological replicate. After 20 d of cold treatment, the rate of change in biomass accumulation, root length, and root number were measured for each plant. The values for the three plants in each pot were averaged to obtain one biological replicate, and the mean of the three replicate pots was calculated for each accession and used for subsequent statistical analyses.

The raw phenotypic data were standardized prior to principal component analysis (PCA). The first principal component (PC1) was extracted to calculate the comprehensive cold-tolerance phenotype score (CTPS) for each accession according to the following equation: Y = 0.314 × biomass change + 0.673 × root length + 0.669 × root number. Based on their CTPS values, the evaluated accessions were classified into three categories: cold-resistant (R, CTPS > 0.4), moderately cold-resistant (M, −0.2 < CTPS ≤ 0.4), and cold-sensitive (N, CTPS ≤ −0.2).

### 4.8. Transcriptome Sequencing and Differential Expression Analysis

To investigate transcriptional responses to cold stress, one representative accession from each cold-tolerance group was selected: CX55 (‘Ji10216’, cold-resistant, R), CX36 (‘Xu17-10’, moderately cold-resistant, M), and CX65 (‘LongShu601’, cold-sensitive, N). Plants were cultivated under the conditions described in Section 4.1 and subjected to cold treatment at 4 °C. Leaf (L) and tuberous root (T) samples were harvested at 0 h and 24 h post-treatment, with three biological replicates for each time point and tissue type. RNA-seq library construction and sequencing were performed by Guangzhou Gene Denovo Honour Biotechnology Co., Ltd. (Guangzhou, China). Raw sequencing reads were filtered using fastp (v1.3.6) [50] with default parameters to obtain clean reads.

To evaluate the stability and reproducibility of the sequencing data, the gene expression matrix was normalized, and PCA was conducted to assess the consistency among the biological replicates under different conditions. Furthermore, the Pearson correlation coefficients between pairwise samples were calculated based on the transposed expression matrix using the cor function in the R stats package (v4.4.2) to generate a sample correlation matrix.

Differentially expressed genes (DEGs) were identified using the R package DESeq2 (v1.46.0) [51], with the significance thresholds set at a false discovery rate (FDR) < 0.05 and |log2 Fold Change| ≥ 1. For functional enrichment analysis, the sweetpotato KEGG and GO annotations were first processed using eggNOG-mapper (v2.1.15). Based on these annotations, a species-specific OrgDb database was constructed using the R package AnnotationForge (v1.48.0) [52]. Subsequently, functional enrichment analyses of the DEGs were performed using the R package clusterProfiler (v4.14.6) [53] with default parameters.

### 4.9. Weighted Gene Co-Expression Network Analysis (WGCNA)

To identify gene co-expression networks in sweetpotato, a stringent filtering process was first applied to all genes across the 36 samples. This included removing lowly expressed genes (FPKM > 0.1 in less than 20% of samples), retaining the top 10,000 highly variable genes based on variance, and performing median normalization. Weighted gene co-expression network analysis (WGCNA) was performed using the WGCNA R package (v1.74). Based on the scale-free topology criterion, the optimal soft threshold was determined to be 24 (R^2^ = 0.855). An unsigned co-expression network was constructed using this parameter, with the minimum module size set to 30 genes and the module merging threshold set to 0.25. Gene co-expression modules were identified using the dynamic tree-cutting algorithm. Module eigengenes were subsequently correlated with experimental traits (including binary-coded variables for six treatment conditions and two time points) to screen for modules significantly associated with the traits (|r| > 0.5, *p* < 0.01). All the above analyses were performed using the WGCNA package in R.

### 4.10. Identification of Homologous Sequences

The protein sequences of IbMPK3 [29,34] and IbSPF1 [34] have been previously reported. To identify their homologs in sweetpotato, these sequences were aligned against the sweetpotato proteome using BLASTP (v2.12.0+, https://blast.ncbi.nlm.nih.gov/Blast.cgi, accessed on 24 November 2025). The BLOSUM62 amino acid substitution matrix was utilized, with an E-value threshold of 1 × 10^−5^, gap opening and extension penalties set to 11 and 1, respectively, and low-complexity region filtering disabled. The top five hits with the highest alignment scores were selected as the corresponding homologous sequences.

### 4.11. Quantitative Real-Time PCR (qRT-PCR) Validation of Homologous Sequences

To validate the expression level of *IbWRKY460* (Ibat.Brg.15D_G008070) in sweetpotato, quantitative real-time PCR (qRT-PCR) was performed. Total RNA was extracted using the RNAprep Pure Plant Plus Kit (TIANGEN Biotech, Beijing, China) according to the manufacturer’s instructions. RNA concentration and purity were determined spectrophotometrically (Nano-300 Micro-Spectrophotometer, Hangzhou Allsheng Instruments Co., Ltd., Hangzhou, China), and 1 μg of total RNA was used for first-strand cDNA synthesis.

For reverse transcription, 1 μg of total RNA was used for first-strand cDNA synthesis with the HiScript III RT SuperMix for qPCR (+gDNA wiper) kit (cat. no. R323-01; Vazyme Biotech Co., Ltd., Nanjing, China). Genomic DNA was removed by mixing 1 μg of total RNA with 4 μL of 4× gDNA wiper Mix and RNase-free ddH2O to a final volume of 16 μL, followed by incubation at 42 °C for 2 min. Subsequently, 4 μL of 5× HiScript III qRT SuperMix was added to give a final reaction volume of 20 μL. Reverse transcription was performed at 37 °C for 15 min, followed by enzyme inactivation at 85 °C for 5 s. The resulting cDNA was stored at −20 °C until use.

Quantitative real-time PCR (qPCR) was performed using a LineGene 9600 Plus Real-Time PCR Detection System (FQD-96A; Bioer Technology Co., Ltd., Hangzhou, China) with ChamQ Universal SYBR qPCR Master Mix (cat. no. Q711-02; Vazyme Biotech Co., Ltd., Nanjing, China). Each 20-μL reaction contained 10 μL of 2× ChamQ Universal SYBR qPCR Master Mix, 0.5 μL each of forward and reverse primers (10 μM), 1 μL of cDNA template, and 8 μL of RNase-free ddH2O. The amplification program consisted of an initial denaturation at 95 °C for 30 s, followed by 40 cycles of 95 °C for 10 s and 61 °C for 30 s. A melting-curve analysis was performed immediately after amplification to verify product specificity. The reporting of the qPCR procedure followed the MIQE guidelines [54].

IbACTIN was used as the internal reference gene. No-template controls and no-reverse-transcription controls were included to exclude reagent contamination and genomic DNA interference. Three biological replicates were analyzed, with three technical replicates for each sample. Relative gene expression was calculated using the 2^−ΔΔCt^ method.

### 4.12. Subcellular Localization of the IbWRKY460

To determine the subcellular localization of IbWRKY460, its coding sequence was cloned into the laboratory-maintained plant expression vector pBIN-GFP2 by homologous recombination (ClonExpress II One Step Cloning Kit, Vazyme Biotech, Nanjing, China) [55,56]. The *Nicotiana benthamiana* plants, *Agrobacterium tumefaciens* strain GV3101, and *Escherichia coli* strain DH5α used in this study were maintained in our laboratory. The recombinant plasmid was introduced into *E. coli* DH5α competent cells by heat-shock transformation following Hanahan [57] and verified by sanger sequencing (Beijing Tsingke Biotech, Beijing, China). The sequence-verified plasmid was subsequently introduced into *A. tumefaciens* GV3101 competent cells using the freeze–thaw method described by Holsters et al. [57,58]. *N. benthamiana* plants were grown in a peat-based substrate mixed with vermiculite (3:1, *v*/*v*) in a growth chamber at 25 ± 2 °C and 60–70% relative humidity under a 16 h light/8 h dark photoperiod with a light intensity of approximately 120 μmol m^−2^ s^−1^. Plants were watered every 2–3 days or as required to maintain adequate substrate moisture. Fully expanded leaves from 4- to 5-week-old plants were infiltrated with A. *tumefaciens* suspensions harboring pBIN-GFP2-IbWRKY460 or the empty pBIN-GFP2 vector. After infiltration, the plants were maintained at approximately 25 °C and 70% relative humidity in darkness for 36 h [59,60]. GFP fluorescence in tobacco leaf epidermal cells was then observed using a Zeiss confocal laser scanning microscope (Carl Zeiss Microscopy GmbH, Jena, Germany).

### 4.13. Cold Tolerance Assay to Assess IbWRKY460 in Yeast

To evaluate the cold tolerance conferred by IbWRKY460, the coding sequence of IbWRKY460 was cloned into the yeast expression vector pYES2-NT B to generate pYES2-NT B-IbWRKY460 [61]. The recombinant plasmid and the corresponding empty vector (pYES2-NT B-EV) were separately transformed into *Saccharomyces cerevisiae* strain BY4741 (MATa his3Δ1 leu2Δ0 met15Δ0 ura3Δ0) [62]. Transformants were cultured at 30 °C for 4 days and then subjected to cold treatments at 4, 0, −10, and −20 °C for 24, 48, 72, and 96 h. After treatment, yeast suspensions were serially diluted to 10^0^, 10^−1^, and 10^−2^ and spotted onto synthetic galactose medium lacking uracil (SG-Ura) plates [61]. The SG-Ura medium contained 1.7 g/L yeast nitrogen base without amino acids and ammonium sulfate (Yeast Nitrogen Base, Without Amino Acid and Ammonium Sulfate, Beijing Solarbio Science & Technology, Beijing, China), 5 g/L ammonium sulfate (Ammonium Sulfate, Beijing Solarbio Science & Technology, Beijing, China), 20 g/L galactose(D(+)-Galactose, Beijing Solarbio Science & Technology, Beijing, China), 0.77 g/L dropout supplement lacking uracil (DO Supplement −Ura, Coolaber, Beijing, China), and 17 g/L agar (Agar, Coolaber, Beijing, China). Plates were incubated at 30 °C for 3–4 days before growth phenotypes were recorded and photographed.

## Data Availability

The original contributions presented in the study are included in the article, further inquiries can be directed to the corresponding author.

## Supplementary Materials

The following supporting information can be downloaded at: https://www.mdpi.com/article/10.3390/plants15193020/s1, Figure S1: Phylogenetic tree, conserved motifs, and domain architectures of IbWRKY proteins in sweet potato; Figure S2: Sequence of representative conserved motifs (Motifs 3, 5, 8, and 10) in IbWRKY proteins. Figure S3: PCA and pearson correlation analysis of transcriptome samples; Table S1: Summary of the identified IbWRKY genes and their detailed characteristics; Table S2: Duplication types of each IbWRKY gene; Table S3: Fisher’s exact tests of different groups and duplication types; Table S4: CTPS of 232 germplasm accessions; Table S5: All IbWRKYs among identified DEGs; Table S6: Genes of different modules acquired from the WGCNA analysis; Table S7: The pearson correlation and *p*-value between WGCNA modules and traits; Table S8: Primers used in this study.

## References

[1] Shekhar S., Mishra D., Buragohain A.K., Chakraborty S., Chakraborty N. (2015). Comparative analysis of phytochemicals and nutrient availability in two contrasting cultivars of sweet potato (*Ipomoea batatas* L.). Food Chem..

[2] Mohanraj R., Sivasankar S. (2014). Sweet potato (*Ipomoea batatas* L. Lam)—A valuable medicinal food: A review. J. Med. Food.

[3] Jin R., Yu T., Guo P., Liu M., Pan J., Zhao P., Zhang Q., Zhu X., Wang J., Zhang A. (2022). Comparative transcriptome and interaction protein analysis reveals the mechanism of *IbMPK3*-overexpressing transgenic sweet potato response to low-temperature stress. Genes.

[4] Dietz K.-J., Vogelsang L. (2024). A general concept of quantitative abiotic stress sensing. Trends Plant Sci..

[5] Hao Y., Zong X., Ren P., Qian Y., Fu A. (2021). Basic Helix-Loop-Helix (bHLH) Transcription Factors Regulate a Wide Range of Functions in *Arabidopsis*. Int. J. Mol. Sci..

[6] Liu H., Tang X., Zhang N., Li S., Si H. (2023). Role of bZIP transcription factors in plant salt stress. Int. J. Mol. Sci..

[7] Wang X., Niu Y., Zheng Y. (2021). Multiple functions of MYB transcription factors in abiotic stress responses. Int. J. Mol. Sci..

[8] Diao P., Chen C., Zhang Y., Meng Q., Lv W., Ma N. (2020). The role of NAC transcription factor in plant cold response. Plant Signal. Behav..

[9] Wang Y., Dong B., Wang N., Zheng Z., Yang L., Zhong S., Fang Q., Xiao Z., Zhao H. (2023). A WRKY transcription factor PmWRKY57 from Prunus mume improves cold tolerance in *Arabidopsis thaliana*. Mol. Biotechnol..

[10] Agarwal P., Reddy M., Chikara J. (2011). WRKY: Its structure, evolutionary relationship, DNA-binding selectivity, role in stress tolerance and development of plants. Mol. Biol. Rep..

[11] Javed T., Gao S.-J. (2023). WRKY transcription factors in plant defense. Trends Genet..

[12] Du L., Chen Z. (2000). Identification of genes encoding receptor-like protein kinases as possible targets of pathogen-and salicylic acid-induced WRKY DNA-binding proteins in *Arabidopsis*. Plant J..

[13] Eulgem T., Rushton P.J., Robatzek S., Somssich I.E. (2000). The WRKY superfamily of plant transcription factors. Trends Plant Sci..

[14] Chen F., Hu Y., Vannozzi A., Wu K., Cai H., Qin Y., Mullis A., Lin Z., Zhang L. (2017). The WRKY transcription factor family in model plants and crops. Crit. Rev. Plant Sci..

[15] Rinerson C.I., Rabara R.C., Tripathi P., Shen Q.J., Rushton P.J. (2015). The evolution of WRKY transcription factors. BMC Plant Biol..

[16] Rushton P.J., Somssich I.E., Ringler P., Shen Q.J. (2010). WRKY transcription factors. Trends Plant Sci..

[17] Ishiguro S., Nakamura K. (1994). Characterization of a cDNA encoding a novel DNA-binding protein, SPF1, that recognizes SP8 sequences in the 5′ upstream regions of genes coding for sporamin and β-amylase from sweet potato. Mol. Gen. Genet. MGG.

[18] Jiang J., Ma S., Ye N., Jiang M., Cao J., Zhang J. (2017). WRKY transcription factors in plant responses to stresses. J. Integr. Plant Biol..

[19] Zhang M., Zhao R., Huang K., Huang S., Wang H., Wei Z., Li Z., Bian M., Jiang W., Wu T. (2022). The OsWRKY63-OsWRKY76-OsDREB1B module regulates chilling tolerance in rice. Plant J..

[20] Kim C., Vo K., Nguyen C., Jeong D., Lee S., Kumar M., Kim S., Park S., Kim J., Jeon J. (2016). Functional analysis of a cold-responsive rice WRKY gene, *OsWRKY71*. Plant Biotechnol. Rep..

[21] Qu J., Xiao P., Zhao Z.Q., Wang Y.L., Zeng Y.K., Zeng X., Liu J.H. (2024). Genome-wide identification, expression analysis of WRKY transcription factors in *Citrus ichangensis* and functional validation of CiWRKY31 in response to cold stress. BMC Plant Biol..

[22] Wang Y., Shu Z., Wang W., Jiang X., Li D., Pan J., Li X. (2016). *CsWRKY2*, a novel WRKY gene from Camellia sinensis, is involved in cold and drought stress responses. Biol. Plant..

[23] Li S., Guo M., Hong W., Li M., Zhu X., Guo C., Shu Y. (2025). Overexpression of a White Clover WRKY Transcription Factor Improves Cold Tolerance in *Arabidopsis*. Agronomy.

[24] Wang Y., Zhuang K., Meng Q., Meng C. (2020). Characterization and analysis of some chilling-response WRKY transcription factors in tomato. Plant Physiol. J..

[25] Wang L., Chen H., Chen G., Luo G., Shen X., Ouyang B., Bie Z. (2024). Transcription factor SlWRKY50 enhances cold tolerance in tomato by activating the jasmonic acid signaling. Plant Physiol..

[26] Guo M., Yang F., Liu C., Zou J., Qi Z., Fotopoulos V., Lu G., Yu J., Zhou J. (2022). A single-nucleotide polymorphism in *WRKY33* promoter is associated with the cold sensitivity in cultivated tomato. New Phytol..

[27] Wang Y., Zhang M., Wu C., Chen C., Meng L., Zhang G., Zhuang K., Shi Q. (2024). SlWRKY51 regulates proline content to enhance chilling tolerance in tomato. Plant Cell Environ..

[28] Kang Z., Meng X., Zhang Y., Chen X., Zheng H., Yuan M., Hu X. (2025). The SlWRKY54-SlZF61 Module Synergistically Regulates SLDLO2 to Enhance Cold Tolerance in Tomato Under Low-Temperature Stress. Plant Cell Environ..

[29] Jin R., Kim H.S., Yu T., Liu M., Yu W., Zhao P., Zhang A., Zhang Q., Liu Y., Cao Q. (2022). Overexpression of *IbMPK3* increases low-temperature tolerance in transgenic sweetpotato. Plant Biotechnol. Rep..

[30] Pan R., Buitrago S., Peng Y., Abou-Elwafa S.F., Wan K., Liu Y., Wang R., Yang X., Zhang W. (2022). Genome-wide identification of cold-tolerance genes and functional analysis of *IbbHLH116* gene in sweet potato. Gene.

[31] Dong W., Tang L., Peng Y., Qin Y., Lin Y., Xiong X., Hu X. (2022). Comparative transcriptome analysis of purple-fleshed sweet potato and its yellow-fleshed mutant provides insight into the transcription factors involved in anthocyanin biosynthesis in tuberous root. Front. Plant Sci..

[32] Cheng L.-r., Han Q.-y., Li Y.-h., Qiu Z.-l., Hou M., Lou B.-g. (2024). Evaluation of resistance to foot rot and analysis of disease resistance related indexes of 60 sweet potato germplasm resources. Acta Agric. Zhejiangensis.

[33] Wu J., Su Y., Pan Z., Wang Y., Zhang Y., Li L., Jiang J., Cao X. (2024). Identification of WRKY transcription factors in *Ipomoea pes-caprae* and functional role of IpWRKY16 in sweet potato salt stress response. BMC Plant Biol..

[34] Park S.-U., Jung Y.J., Kwon H.J., Lee J.Y., An J., Lee H.-U., Jeong J.C., Bian X., Yu T., Kwak S.-S. (2025). IbMPK3/IbMPK6-mediated IbSPF1 phosphorylation promotes cold stress tolerance in sweet potato. Biochem. Biophys. Res. Commun..

[35] Sasaki K., Imai R. (2012). Pleiotropic Roles of Cold Shock Domain Proteins in Plants. Front. Plant Sci..

[36] Kim M.-H., Sasaki K., Imai R. (2009). Cold Shock Domain Protein 3 Regulates Freezing Tolerance in *Arabidopsis thaliana*. J. Biol. Chem..

[37] Jiao C., Sun J., Wei Y. (2024). SlWRKY31 enhances chilling tolerance by interacting with SlSIZ1 in tomato fruit. Postharvest Biol. Technol..

[38] Huang S., Gao Y., Liu J., Peng X., Niu X., Fei Z., Cao S., Liu Y. (2012). Genome-wide analysis of WRKY transcription factors in *Solanum lycopersicum*. Mol. Genet. Genom..

[39] Yan M., Li M., Wang Y., Wang X., Moeinzadeh M.H., Quispe-Huamanquispe D.G., Fan W., Fang Y., Wang Y., Nie H. (2024). Haplotype-based phylogenetic analysis and population genomics uncover the origin and domestication of sweetpotato. Mol. Plant.

[40] Yang J., Moeinzadeh M.H., Kuhl H., Helmuth J., Xiao P., Haas S., Liu G., Zheng J., Sun Z., Fan W. (2017). Haplotype-resolved sweet potato genome traces back its hexaploidization history. Nat. Plants.

[41] Liu S., Zhang C., Guo F., Sun Q., Yu J., Dong T., Wang X., Song W., Li Z., Meng X. (2022). A systematical genome-wide analysis and screening of WRKY transcription factor family engaged in abiotic stress response in sweetpotato. BMC Plant Biol..

[42] Pan J., Yang Z., Liu S., Liu Z., Yu T. (2026). The bHLH Transcription Factor IbbHLH129 Positively Regulates the Cold Tolerance of Sweetpotato Seedlings by Modulating Auxin and Gibberellin Pathways. Plants.

[43] Blum M., Andreeva A., Florentino L.C., Chuguransky S.R., Grego T., Hobbs E., Pinto B.L., Orr A., Paysan-Lafosse T., Ponamareva I. (2025). InterPro: The protein sequence classification resource in 2025. Nucleic Acids Res..

[44] Paysan-Lafosse T., Andreeva A., Blum M., Chuguransky S.R., Grego T., Pinto B.L., Salazar G.A., Bileschi M.L., Llinares-López F., Meng-Papaxanthos L. (2025). The Pfam protein families database: Embracing AI/ML. Nucleic Acids Res..

[45] Edgar R.C. (2022). Muscle5: High-accuracy alignment ensembles enable unbiased assessments of sequence homology and phylogeny. Nat. Commun..

[46] Wong T.K.F., Ly-Trong N., Ren H., Demotte P., Baños H., Roger A.J., Susko E., Bielow C., De Maio N., Goldman N. (2026). IQ-TREE 3: Phylogenomic inference software using complex evolutionary models. Mol. Biol. Evol..

[47] Feng J., Chen C., Wu Y., Fan L., Zhang Z., Chen M., Tang H., Yu G., Ren J., Luo J. (2026). How to make big data accessible to plant biologists and beyond: Ten years of lessons from TBtools. Mol. Plant.

[48] Pertea G., Rozenberg A., Maarten-vd-Sande (2021). gpertea/gffread: v0.12.7.

[49] Wang Y., Tang H., DeBarry J.D., Tan X., Li J., Wang X., Lee T.-H., Jin H., Marler B., Guo H. (2012). MCScanX: A toolkit for detection and evolutionary analysis of gene synteny and collinearity. Nucleic Acids Res..

[50] Chen S. (2025). fastp 1.0: An ultra-fast all-round tool for FASTQ data quality control and preprocessing. iMeta.

[51] Love M.I., Huber W., Anders S. (2014). Moderated estimation of fold change and dispersion for RNA-seq data with DESeq2. Genome Biol..

[52] Carlson M., Pagès H. (2024). AnnotationForge: Tools for Building SQLite-Based Annotation Data Packages.

[53] Xu S., Hu E., Cai Y., Xie Z., Luo X., Zhan L., Tang W., Wang Q., Liu B., Wang R. (2024). Using clusterProfiler to characterize multiomics data. Nat. Protoc..

[54] Bustin S.A., Benes V., Garson J.A., Hellemans J., Huggett J., Kubista M., Mueller R., Nolan T., Pfaffl M.W., Shipley G.L. (2009). The MIQE Guidelines: Minimum Information for Publication of Quantitative Real-Time PCR Experiments. Clin. Chem..

[55] Cheng Y., Meng R., Niu S., Peng H., Jing M. (2024). Protocol for quantitative evaluation of misfolded protein degradation using *Agrobacterium*-mediated expression system in *Nicotiana benthamiana*. STAR Protoc..

[56] Bevan M. (1984). Binary Agrobacterium vectors for plant transformation. Nucleic Acids Res..

[57] Hanahan D. (1983). Studies on transformation of Escherichia coli with plasmids. J. Mol. Biol..

[58] Holsters M., de Waele D., Depicker A., Messens E., van Montagu M., Schell J. (1978). Transfection and transformation of *Agrobacterium tumefaciens*. Mol. Gen. Genet. MGG.

[59] Sparkes I.A., Runions J., Kearns A., Hawes C. (2006). Rapid, transient expression of fluorescent fusion proteins in tobacco plants and generation of stably transformed plants. Nat. Protoc..

[60] Leuzinger K., Dent M., Hurtado J., Stahnke J., Lai H., Zhou X., Chen Q. (2013). Efficient agroinfiltration of plants for high-level transient expression of recombinant proteins. JoVE J. Vis. Exp..

[61] Life Technologies Corporation (2012). pYES2/NT A, B, and C and pYC2/NT A, B, and C: Yeast Expression Vectors with N-Terminal Tags.

[62] Baker Brachmann C., Davies A., Cost G.J., Caputo E., Li J., Hieter P., Boeke J.D. (1998). Designer deletion strains derived from Saccharomyces cerevisiae S288C: A useful set of strains and plasmids for PCR-mediated gene disruption and other applications. Yeast.

