# Supplementary material for: Genome-Wide Identification and Functional Validation of the Cold-Tolerance WRKY Transcription Factor in Sweetpotato
Source: Plants (Basel). 2026 Oct 2;15(19):3020. doi: 10.3390/plants15193020 (PMC13666874; doi:10.3390/plants15193020)
Supplement: Supplementary file 1 [file plants-15-03020-s001.zip › Supplementary Figures.pdf]

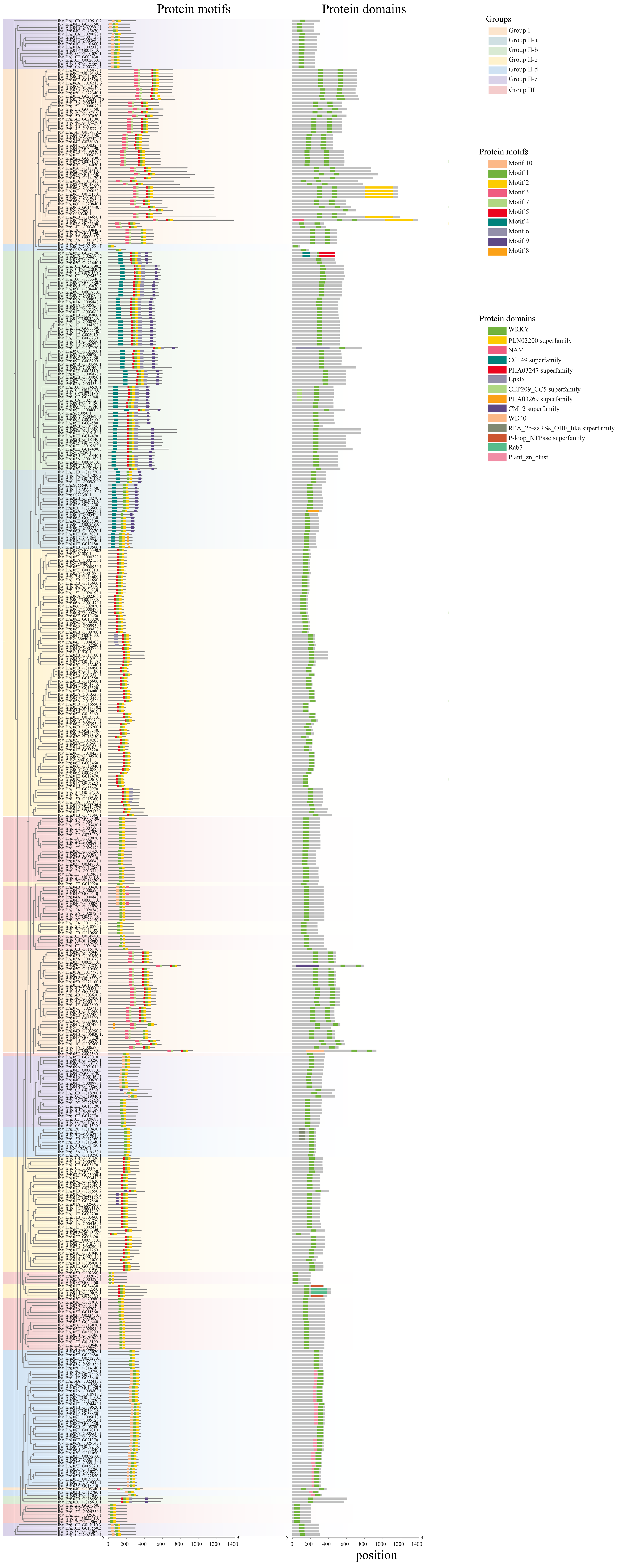

Figure S1. Phylogenetic tree, conserved motifs, and domain architectures of IbWRKY proteins in sweet potato.
Maximum likelihood (ML) phylogenetic tree of IbWRKY proteins with background colors indicating different subfamilies, displayed alongside the MEME-predicted conserved motifs and CDD-identified protein domain architectures, with the scale bar representing protein length in amino acids (aa).

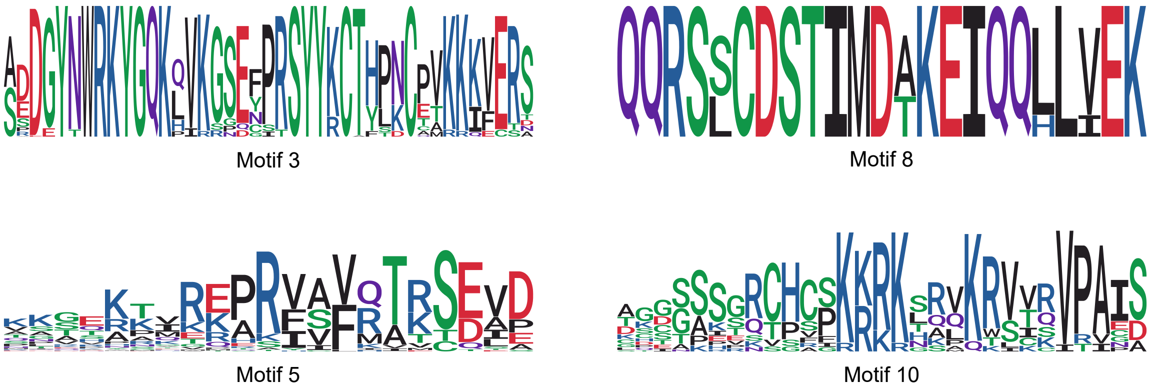

Figure S2. Sequence of representative conserved motifs (Motifs 3, 5, 8, and 10) in IbWRKY proteins. Predicted by the MEME suite, where the height of each amino acid letter reflects its degree of conservation at that position, and different colors indicate distinct chemical properties of the amino acid residues.

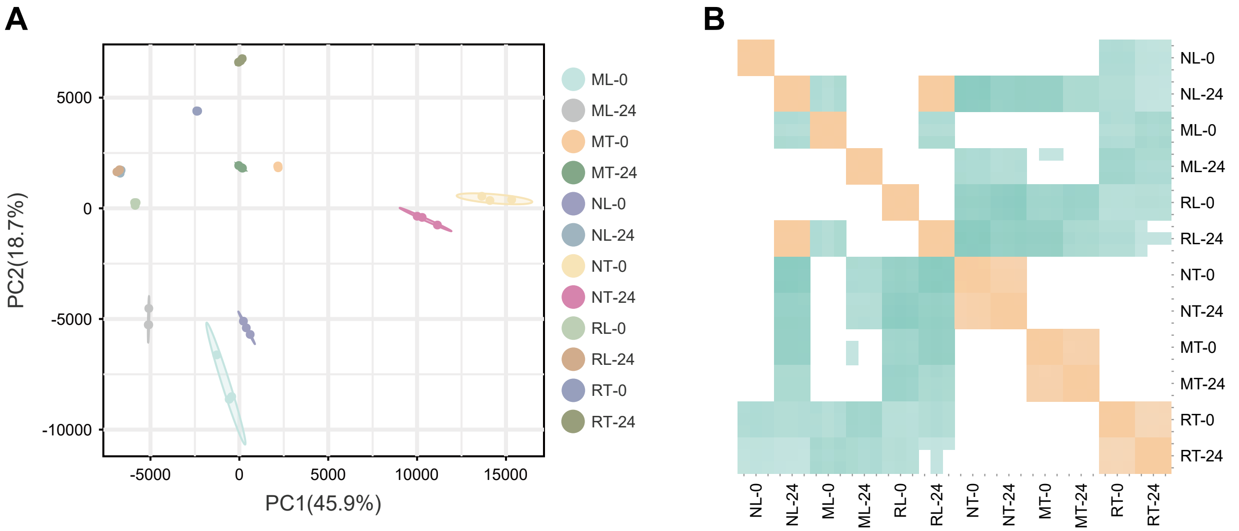

Figure S3. PCA and pearson correlation analysis of transcriptome samples. A:Principal component analysis (PCA) of gene expression profiles in leaves (L) and roots (T) of R, M, and N accessions under control (0 h) and cold stress (24 h) conditions. B:Pearson correlation heatmap among all sample replicates based on gene expression levels.
